# Supracondylar Humeral Fractures: An Audit of the Frequency of Bi-columnar Fixation and Intra-articular Wire Placement

**DOI:** 10.7759/cureus.2318

**Published:** 2018-03-13

**Authors:** Andrew Ker, Claire Murnaghan, James S Huntley

**Affiliations:** 1 Paediatric Orthopaedic Department, RHC Glasgow; 2 Department of Surgery, Sidra Medicine

**Keywords:** pediatric supracondylar humerus fracture, percutaneous wire fixation

## Abstract

Introduction

For supracondylar humeral (SCH) fractures, biomechanical studies suggest the most stable wire configuration achieves bi-columnar fixation. Achieving medial column fixation using lateral-entry-only wires may require an intracapsular entry point. The aim of this study was to identify the rate of bi-columnar fixation achieved in our department when treating SCH fractures with percutaneous wire fixation. A secondary aim was to identify the rate of placement of an intra-articular wire. Further aims were to examine if failure to achieve bi-columnar fixation was associated with an increased loss of fixation and whether the placement of an intra-articular wire resulted in any cases of deep infection or septic arthritis.

Material and methods

All Gartland type 3 supracondylar humeral fractures, June 2014 to December 2016, were retrospectively identified. Intra-operative films were reviewed to determine bi-columnar fixation and the presence/absence of an intra-articular wire. Loss of reduction requiring revision and post-operative infections were determined from the electronic patient record.

Results

Of 49 supracondylar fractures identified, 42 were fixed with lateral-entry only wires (24 with two wires and 18 with three wires), and seven were fixed with medial/lateral cross wires (four with one lateral wire, two with two wires, and one with three wires). Bi-columnar fixation was achieved in 41/49 cases (84%). All cases where bi-columnar fixation was not achieved were fixed with lateral-entry-only wires. One out of 49 fractures (2%) required the revision of fixation at 10 days due to loss of reduction. In this case, the initial fixation was with two lateral-entry-only wires, without bi-columnar fixation. An intra-articular wire was present in 44 out of 49 cases (90%). One out of 49 cases (2%) had a superficial wound infection. There were no cases of deep infection or septic arthritis.

Conclusion

In our department, the rate of bi-columnar fixation was high and, in this group, no cases required revision fixation. One of eight cases judged to not have bi-columnar fixation initially, required revision due to loss of fixation. We contend that bi-columnar fixation generally achieves a stable wire configuration even using lateral-entry-only wires for SCH fractures. The rate of intra-articular wire placement was high; however, infection rates were low with no cases of septic arthritis.

## Introduction

The supracondylar humeral (SCH) fracture is the most common pediatric fracture near the elbow [1]. Gartland’s classification [2] of extension-type SCH fractures was modified by Wilkins [3] into four types: nondisplaced (type 1), displaced with an intact posterior cortical hinge and no rotational displacement (type 2A), displaced with posterior cortical hinge with rotational displacement (type 2B) and completely displaced fractures (type 3). Historically, SCH fractures were treated with closed reduction and cast immobilization [4]. For minimally displaced, more stable fractures (types 1 and 2A), this remains a satisfactory method [5]. However, this approach has largely been abandoned in the treatment of displaced SCH fractures (types 2B and 3) due to the difficulties in maintaining reduction and circulation [6-8].

The percutaneous wire fixation of SCH fractures, described by Swenson in 1948 [9], has evolved and is now considered the “Gold Standard” for displaced fractures [10]. Current UK National guidelines state that all displaced SCH fractures should be treated by “early surgical stabilization” with “bicortical wire fixation” [11]. Two basic techniques have been described: medial/lateral cross wires and lateral-entry-only wires. In vitro biomechanical studies suggest that medial/lateral cross wires provide more stability than using lateral-entry-only wires [12-13]. However, there are equivocal results when assessing stability between the two techniques in clinical trials [14-17]. Also, the risk of iatrogenic ulnar nerve injury is greater when using cross wires [17-18].

**Figure 1:**
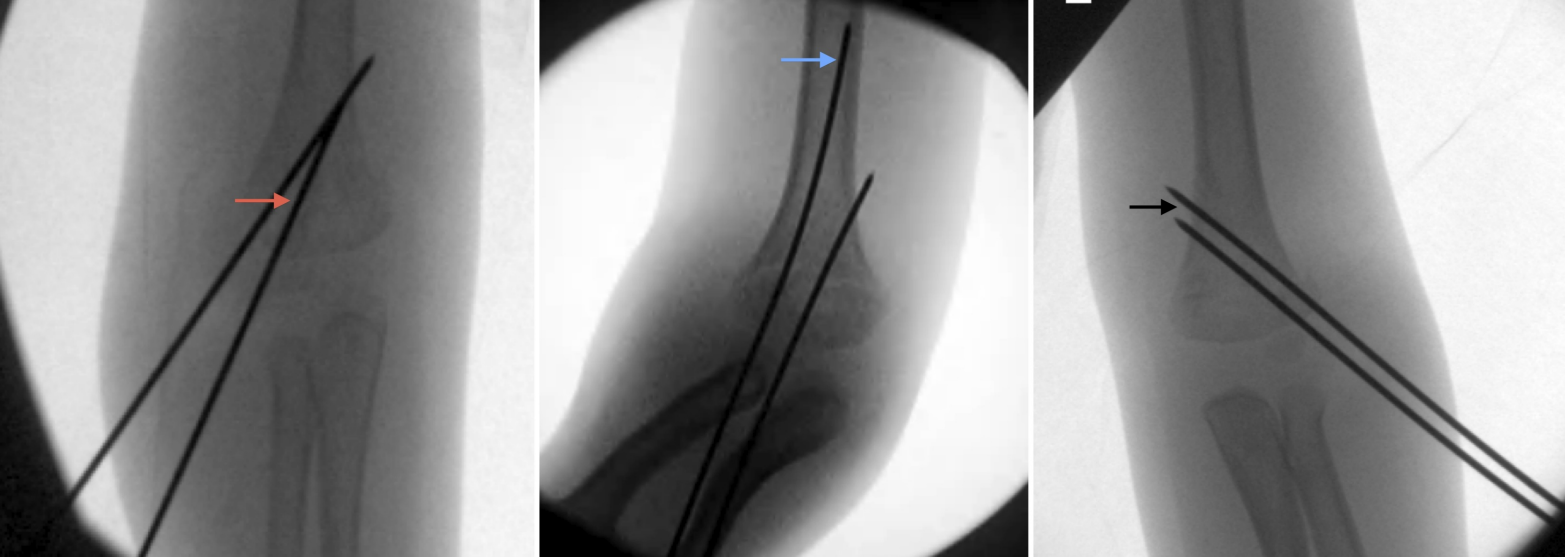
Type A error: failure to engage the distal fragment (red arrow); Type B error: failure to achieve bi-cortical purchase (blue arrow); Type C error: inadequate separation of wires (black arrow); as identified by Sankar et al.

For lateral-entry-only wires, biomechanical studies suggest that both the divergence of wires and the sufficient separation of wires at the fracture site are associated with stability [12,19]. Sankar et al. [20] identified other surgical errors increasing the risk of loss of fixation, including: (i) failure to engage the distal fragment; (ii) failure to achieve bicortical purchase; and (iii) inadequate separation of wires (Figure 1).

The concept of bi-columnar fixation was recently elaborated in unpublished work by Jones et al., achieving a stable configuration by ensuring the wire fixation of both the medial and lateral columns of the distal humerus and thereby obviating the technical errors described by Sankar et al. [20].

The risk of a pin-track infection following the percutaneous wire fixation of SCH fractures varies from zero to 8% [21-24]. The majority are superficial infections that result in no long-term sequelae; septic arthritis and osteomyelitis are rare. However, several studies assessing optimal wire placement using lateral-entry-only wires have recommended using extra-articular divergent wires to decrease the potential for septic arthritis [25].

The aim of this study was to identify the rate of bi-columnar fixation achieved in our department when treating SCH fractures with percutaneous wire fixation. A secondary aim was to identify the rate of placement of an intra-articular wire. Further secondary aims were to examine if failure to achieve bi-columnar fixation was associated with an increased loss of fixation and whether placement of an intra-articular wire resulted in any cases of deep infection or septic arthritis.

## Materials and methods

Population and patients

We retrospectively identified patients presenting to the Royal Hospital for Children, Glasgow, with an SCH fracture between April 2014 and December 2016, using the local database of trauma admissions. Using the patient’s unique identifier number, their online operation note was reviewed to confirm that they had undergone an operation to treat an SCH fracture in our department within the given dates. The lead author used the national picture archiving and communication system (PACS) software and database of patient records to identify patients eligible for the study.

Inclusion/exclusion criteria

All fractures were classified using the Wilkins modification [3] of the Gartland classification [2]. All patients that had undergone wire fixation for a completely displaced extension-type SCH fracture (type 3 fracture) or flexion-type SCH fracture were included. Fractures treated with closed or open reduction and all wire configurations (lateral-entry-only or medial/lateral cross-wires) were included.

Patients with a minimally displaced fracture or extension-type fractures with an intact posterior cortical hinge (Gartland type 1 and 2 A or B fractures) were excluded. The patient was excluded if the initial operation had been performed at a different hospital or if follow-up notes or radiographs to the point of discharge from the outpatient clinic were not available. Forty-nine patients (20 boys and 29 girls with a mean age of eight years old; range: two to 14 years) met the inclusion criteria. One hundred and fifteen patients were excluded; 110 patients with Gartland types 1 and 2 A or B fractures and five patients who were followed up in other hospitals.

Operative technique

All had a general anesthetic and a single prophylactic dose of cefuroxime at induction. An image intensifier was used to confirm reduction and wire position intra-operatively. In 48 out of 49 cases, closed reduction was achieved successfully. One case required open reduction via an anterior approach. Fixation was achieved using 2.0 mm Kirschner wires in 32 patients and 1.6 mm Kirschner wires in seven patients. In the remaining 10 patients, the diameter of wires used was not mentioned in the operation note. Following fixation, wires were left proud of the skin and patients placed in an above-elbow backslab cast with the elbow at 90 degrees of flexion and the forearm in neutral rotation. Post-operatively, the backslab was reinforced to a full cast prior to discharge.

Subsequent management

Patients had a radiograph one-week post-operatively, depending on the surgeon’s preference, and all had a radiograph at three to four weeks when the wires and cast were removed followed by mobilization. All patients were either reviewed or given an optional appointment for review at six weeks following mobilization.

Primary outcome measures

In all patients, we recorded whether bi-columnar wire fixation was achieved and whether there was intra-articular wire placement.

**Figure 2:**
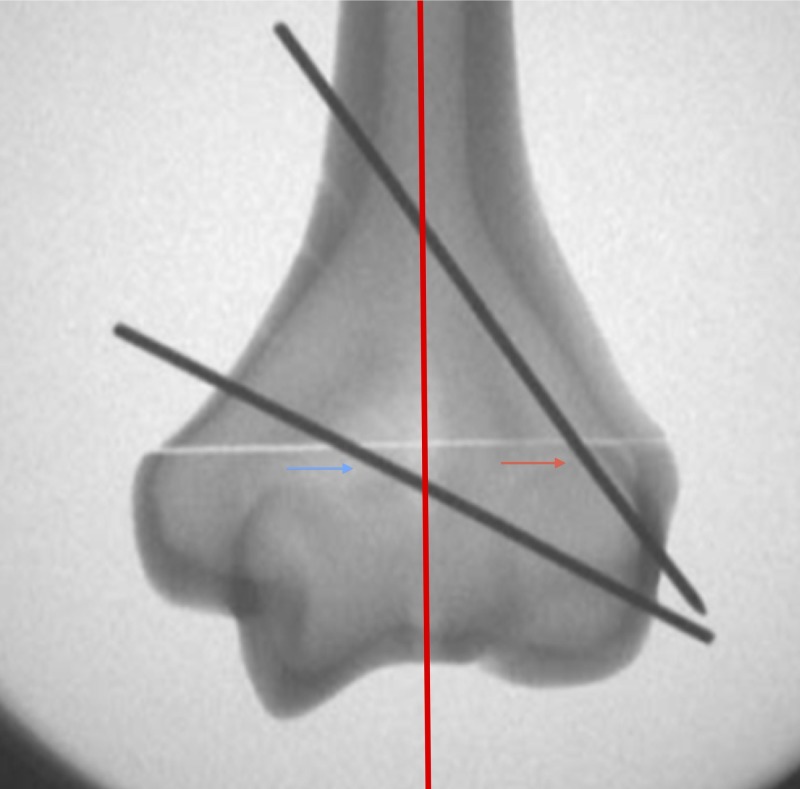
Assessment of AP intraoperative radiograph for bi-columnar fixation. Distal humerus divided into medial and lateral columns (as indicated by the red line). Bi-columnar fixation achieved if (1) a wire is traversing the lateral half of the distal fragment (red arrow); (2) a wire is traversing the medial half of the distal fragment (blue arrow); and (3) both wires have purchase in the medial and lateral cortices. AP: anteroposterior

Bi-columnar fixation was assessed on the intra-operative anteroposterior (AP) radiograph by drawing a straight line down the center of the humeral shaft, parallel to the humeral shaft, dividing the distal humerus into medial and lateral columns. Bi-columnar fixation was achieved only if there was (1) a wire traversing the lateral half of the distal fragment; (2) a wire traversing the medial half of the distal fragment; and (3) both wires had purchase in medial and lateral cortices (Figure 2).

We used the methods described in a recent cadaveric study combined with imaging to identify sites of intra-articular wire placement [24]. Laterally placed wires were extra-articular if, on the intra-operative anteroposterior radiograph, the wire starting point was on the lateral side of the capitellum, proximal to the mid-waist, and on the lateral radiograph, the entry point was within the cortical outlines of the “hour-glass” of the distal humerus. Medially placed wires were extraarticular if the entry point was within the medial epicondyle on the anteroposterior (AP) image.

Secondary outcome measures

These were the maintenance/loss of fixation and occurrence/absence of infection. The loss of fixation was determined by a review of follow-up radiographs by the lead author. The rate of infection was determined by a review of post-operative notes up until the point of discharge from the follow-up outpatient clinic. Post-operative notes and imaging were available for all patients and no patients were lost to follow-up.

## Results

All 49 Gartland type 3 SCH fractures identified were operated on by one of nine consultant surgeons working within the department over the study period or by a specialty trainee or clinical fellow under the supervision of the consultant who was present in the theater. There were 46 extension-type fractures (94%) and three flexion-type fractures (6%).

Wire configurations

Forty-two of 49 patients (86%) were treated with lateral-entry-only wires. Conversely, seven out of 49 patients (14%) were treated with medial/lateral cross wires, with a varying number of lateral wires used (Table 1).

**Table 1:** Configuration of wires used

Lateral-Entry-Only Wires		Medial and Lateral-Entry Wires			Total
2 Lateral Wires	3 Lateral Wires	1 Medial and 1 Lateral Wire	1 Medial and 2 Lateral Wires	1 Medial and 3 Lateral Wires	
24	18	4	2	1	49

Bi-columnar fixation

In 41 out of 49 cases (84%), bi-columnar fixation was achieved. All seven patients that had medial/lateral cross wires had bi-columnar fixation (100%). Of the patients that had lateral-entry-only wires, 34 out of 42 cases (81%) had bi-columnar fixation.

**Figure 3:**
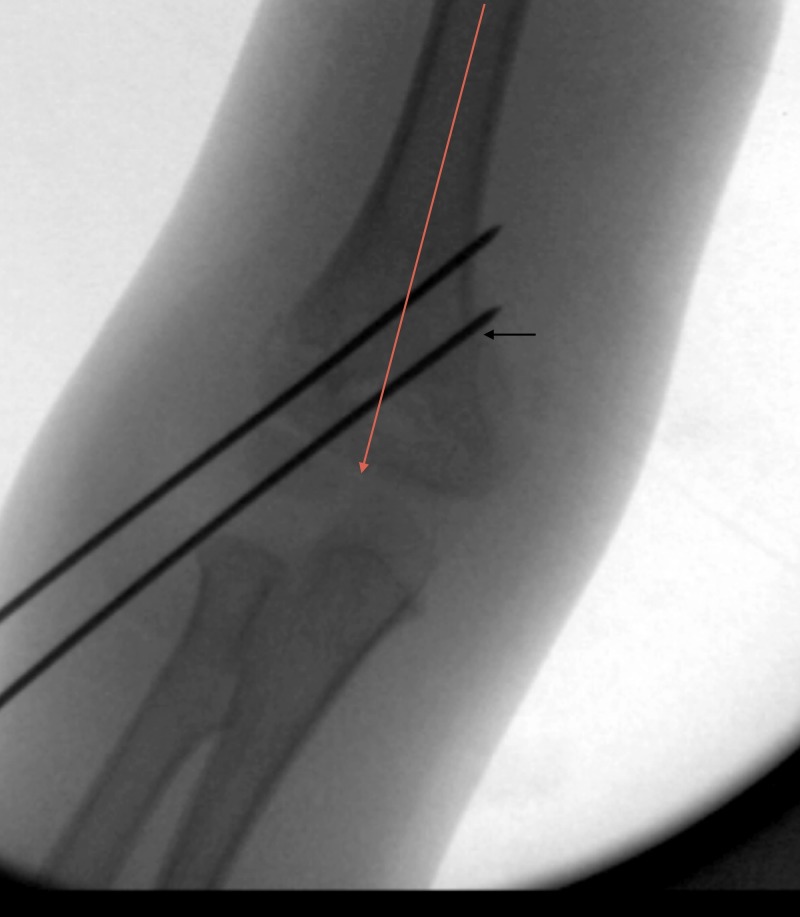
Intraoperative AP radiograph demonstrating fixation without bi-columnar fixation. The lateral wire (black arrow) does not have purchase in the lateral column distally. AP: anteroposterior

**Figure 4:**
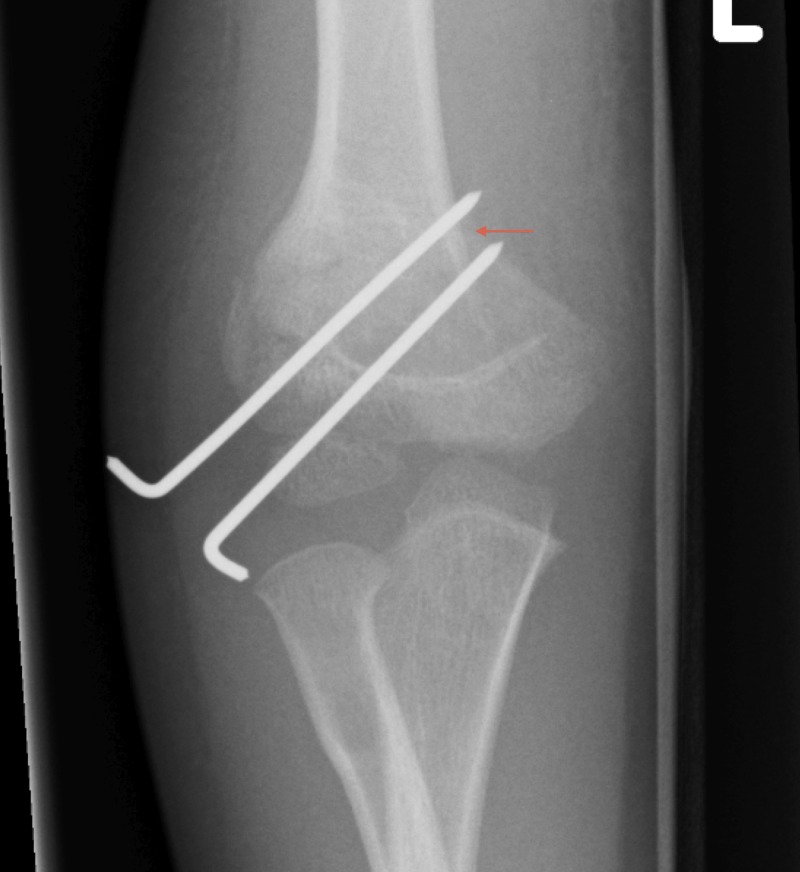
Failure of fixation identified on one-week follow-up radiograph (red arrow).

There was one case of loss of fixation requiring revision fixation (2%). This occurred in one of the eight patients (13%) where bi-columnar fixation was not achieved on the intra-operative screening radiographs. In this case, the patient underwent closed reduction and fixation with two lateral-entry-only wires (Figure 3). Loss of fixation was identified in the one-week post-operative check radiographs and the patient underwent a further closed reduction and revision fixation with medial/lateral cross wires (Figure 4). There were no episodes of loss of fixation in all 41 patients that were initially treated with bi-columnar fixation (Table 2).

**Table 2:** Loss of fixation requiring revision fixation

Bicolumnar Fixation Achieved	Bicolumnar Fixation NOT Achieved	Total
0/41	1/8 (13%)	1/49 (2%)

Intra-articular wire placement

In 44 out of 49 cases (90%), there was the placement of an intra-articular wire. All episodes of intra-articular wire placement occurred with lateral-entry wires and no medial wires were placed intra-articularly. Of the five cases where there were no intra-articular wires, two patients did not have bi-columnar fixation (40%). In the 44 cases where there was an intra-articular wire, six patients did not have bi-columnar fixation (13%). There was one superficial infection (2%) that was treated successfully with a single-week course of oral antibiotics. There were no cases of deep infection or septic arthritis.

Variability between surgeons within the department

There was considerable variation in the rate of bi-columnar fixation achieved between different surgeons within the department. Four out of nine surgeons achieved bi-columnar fixation 100 percent of the time. There was less variability when comparing the rate of intra-articular wire placement, with seven out of nine surgeons placing an intra-articular wire 100 percent of the time (Table 3).

**Table 3:** Variability between surgeons within the department in the rate of bi-columnar fixation and intra-articular wire placement

Surgeon	Number of Procedures	Bi-columnar Fixation	Intra-articular Wire Placement
1	9	8 (89%)	9 (100%)
2	15	12 (80%)	15 (100%)
3	4	4 (100%)	4 (100%)
4	4	4 (100%)	1 (25%)
5	7	6 (86%)	7 (100%)
6	1	0	1 (100%)
7	5	3 (60%)	2 (40%)
8	2	2 (100%)	2 (100%)
9	2	2 (100%)	2 (100%)

## Discussion

In managing SCH fractures in children, the aim of treatment is to gain a functional and cosmetically acceptable upper limb with a normal range of movement [1]. For displaced SCH fractures, closed or open reduction, if required, followed by wire fixation is widely accepted as the optimal treatment and current national guidelines state that these fractures be managed by reduction and wire fixation [11].

There is conflicting evidence in the literature regarding the stability of SCH fracture fixation when comparing medial/lateral cross wires and lateral-entry-only wires. A number of in vitro biomechanical studies have shown medial/lateral cross wires to be more stable [12-13]. A systematic review of clinical trials carried out by Brauer et al. [26] in 2007, including 35 studies (two randomized control trials), found that medial/lateral cross wires were a more stable configuration with reduced risk of loss of fixation. However, a number of clinical studies have found no difference in terms of loss of fixation between medial/lateral cross wires and lateral-entry-only wire configurations [14-17]. Sibinski et al [14] reviewed 131 patients (65 treated with cross wires and six with lateral-entry-only wires) and found no statistical difference in the overall radiological and clinical outcomes between the two groups. A recent systematic review by Yousri et al. [27] in 2014, identified four randomized control trials and found no significant difference in terms of loss of reduction between the two groups, suggesting similar stability for both constructs.

Although a medial/lateral cross wire configuration potentially provides a more stable construct, the main disadvantage is the risk of ulnar nerve injury during the placement of the medial wire [17-18]. A systematic review in 2010, comprising 32 studies of 2639 patients, comparing medial/lateral cross wires against lateral-entry-only wires, found a significantly increased incidence of iatrogenic ulnar nerve injury in the cross-wire group (risk difference 0.035), with a suggested number needed to harm of 28 [18]. A meta-analysis in 2012, including 18 studies, found the risk of iatrogenic ulnar nerve injury to be 4.3 times higher for patients treated with cross wiring [17].

Due to the increased risk of iatrogenic ulnar nerve injury, many surgeons prefer to use a lateral-entry-only wire technique. In vitro biomechanical studies have shown that both the divergence of wires and the increased separation of wires at the fracture site are more stable and have attributed the strength of divergent wires to an increased purchase in the medial and lateral columns of the distal humerus [12,19]. A large clinical study concerning the loss of fixation and wire configuration retrospectively reviewed 279 patients and reported eight (2.9%) episodes of postoperative loss of fixation [20]. All of these were in Gartland type 3 fractures; seven treated with two lateral entry wires and one treated with medial and lateral cross wires. In all cases of loss of fixation, the authors identified technical errors at the time of operation, and they divided them into three types of errors: type A: failure of a wire to engage the distal fragment; type B: failure to achieve bicortical purchase; and type C: failure due to inadequate separation.

Bi-columnar fixation, using the technique described by Jones (unpublished), aims to achieve a biomechanically stable configuration, with bicortical fixation of both medial and lateral columns. This can be achieved using lateral-entry-only wires, thus reducing the risk of iatrogenic ulnar nerve injury.

In our retrospective study, using the technique described to assess the intra-operative AP imaging for bi-columnar fixation, this was achieved in 41 out of 49 cases (84%). Interestingly, in all cases where bi-columnar fixation was achieved, there were no patients with loss of fixation on follow-up radiological evaluation. In contrast, in eight patients who did not have bi-columnar fixation initially, one patient required revision due to loss of initial fixation (13%).

In our department, there was a high rate of intra-articular wire placement when placing lateral-entry-only percutaneous wires, with most surgeons favoring an intra-articular position. There was more marked variability when assessing the rates of bi-columnar fixation with less than half of the surgeons achieving bi-columnar fixation 100 percent of the time. The results of this study, along with current, available literature, have been presented within the department, allowing reflection on current practice and the questionable need for any change in emphasis.

The risk of infection following percutaneous wire fixation of SCH fractures varies from zero to 8% [21-24]. The majority of these are superficial pin-track infections. Parikh et al. [24] reviewed 490 patients and found 21 infections (4.3%) of which six were septic arthritis or osteomyelitis (1.2%). Although our sample size is too small to make conclusions about the risk of septic arthritis with an intra-articular wire, there were no deep infections reported.

Our study has limitations. The assessment of bi-columnar fixation was based on intra-operative radiographs that were not standardized and leaves a potential for error. There is a small sample size, precluding a major statistical analysis. There are other factors that may affect the stability of fixation in treating SCH fractures with percutaneous wires that have not been elaborated on in this study, including the number of wires used, the diameter of wires used, and fracture configuration. Several studies have shown the use of three lateral wires increases fixation strength compared to two lateral wires [16,19]. The diameter of wires used has also been shown to affect the stability of fracture fixation in biomechanical studies [28]. A biomechanical study investigated stability using two lateral-entry-only wires, three lateral-entry-only wires, and cross wires for low transverse, high transverse, and sagittal oblique fractures and found that all wire configurations were rotationally less stable in high transverse fractures compared to the other two fracture configurations [29]. Other studies have used intra-operative fluoroscopic screening to assess dynamic stability during fixation [30]. The advantage of this method is that the wire configuration can be altered intra-operatively.

In the future, we plan to reassess the rates of bi-columnar fixation, intra-articular placement, and infection to see whether there has been a change in practice/outcome. Furthermore, we plan to do an analogous study in other units in Scotland, in particular, nonspecialist pediatric orthopedic units, to assess variability in practice.

## Conclusions

In our study, the rate of bi-columnar fixation was high and, in this group, no cases required revision fixation. One out of eight cases, judged to not have bi-columnar fixation initially, required revision due to loss of fixation. The rate of intra-articular wire placement was high, however, infection rates were low, with no cases of septic arthritis. We contend that bi-columnar fixation generally achieves a stable wire configuration even using lateral-entry-only wires for SCH fractures. This method to assess the adequacy of fixation can easily be used by the operative surgeon intra-operatively using the AP intra-operative radiograph.
